# Epidemiology and risk factors for recurrence in biliary source bloodstream infection episodes in oncological patients

**DOI:** 10.1128/spectrum.02142-23

**Published:** 2023-08-23

**Authors:** Ignacio Grafia, Mariana Chumbita, Elia Seguí, Celia Cardozo, Juan Carlos Laguna, Marta García de Herreros, Nicole Garcia-Pouton, Ana Villaescusa, Cristina Pitart, Verónica Rico-Caballero, Javier Marco-Hernández, Carles Zamora, Margarita Viladot, Joan Padrosa, Albert Tuca, Eric Mayor-Vázquez, Francesc Marco, Jose A. Martínez, Josep Mensa, Carolina Garcia-Vidal, Alex Soriano, Pedro Puerta-Alcalde

**Affiliations:** 1 Medical Oncology Department, Hospital Clínic-IDIBAPS, Barcelona, Spain; 2 Infectious Diseases Department, Hospital Clínic-IDIBAPS, Barcelona, Spain; 3 Microbiology Department, Centre Diagnòstic Biomèdic, Hospital Clínic, Barcelona, Spain; 4 Internal Medicine Department, Supportive and Palliative Care in Cancer Unit, Hospital Clínic, Barcelona, Spain; 5 Medical Intensive Care Unit, Internal Medicine Department, Hospital Clínic-IDIBAPS, Barcelona, Spain; 6 ISGlobal, Hospital Clínic - Universitat de Barcelona, Barcelona, Spain; 7 University of Barcelona, Barcelona, Spain; 8 CIBERINF, CIBER in Infectious Diseases, Barcelona, Spain; Instituto de Investigacion Sanitaria Gregorio Maranon, Madrid, Spain

**Keywords:** cholangitis, biliary source bloodstream infection, mortality, empirical treatment, recurrence

## Abstract

We aimed to describe the characteristics and outcomes of biliary source bloodstream infections (BSIs) in oncological patients. Secondarily, we analyzed risk factors for recurrent BSI episodes. All episodes of biliary source BSIs in oncological patients were prospectively collected (2008–2019) and retrospectively analyzed. Logistic regression analyses were performed. A rule to stratify patients into risk groups for recurrent biliary source BSI was conducted. Four hundred biliary source BSIs were documented in 291 oncological patients. The most frequent causative agents were *Escherichia coli* (42%) and *Klebsiella* spp. (27%), and 86 (21.5%) episodes were caused by multidrug-resistant Gram-negative bacilli (MDR-GNB). The rates of MDR-GNB increased over time. Overall, 73 patients developed 118 recurrent BSI episodes. Independent risk factors for recurrent BSI episodes were prior antibiotic therapy (OR 3.781, 95% CI 1.906–7.503), biliary prosthesis (OR 2.232, 95% CI 1.157–4.305), prior admission due to suspected biliary source infection (OR 4.409, 95% CI 2.338–8.311), and BSI episode caused by an MDR-GNB (OR 2.857, 95% CI 1.389–5.874). With these variables, a score was generated that predicted recurrent biliary source BSI with an area under the receiver operating characteristic (ROC) curve of 0.819. Inappropriate empirical antibiotic treatment (IEAT) was administered in 23.8% of patients, and 30-d mortality was 19.5%. As a conclusion, biliary source BSI in oncological patients is mainly caused by GNB, with high and increasing MDR rates, frequent IEAT, and high mortality. Recurrent BSI episodes are frequent. A simple score to identify recurrent episodes was developed to potentially establish prophylactic strategies.

**IMPORTANCE:**

This study shows that biliary source bloodstream infections (BSIs) in oncological patients are mainly caused by Gram-negative bacilli (GNB), with high and increasing rates of multidrug resistance. Importantly, recurrent biliary source BSI episodes were very frequent and associated with delays in chemotherapy, high rates of inappropriate empirical antibiotic therapy, and high 30-d mortality (19.5%). Using the variable independently associated with recurrent BSI episodes, a score was generated that predicted recurrent biliary source BSI with high accuracy. This score could be used to establish prophylactic strategies and lower the risk of relapsing episodes and the associated morbidity and mortality.

## INTRODUCTION

Biliary source bloodstream infection (BSI) is a frequent infectious complication in oncological patients due to local or metastatic compromise of the biliary tract (1 – 3), causing significant morbidity and mortality.

In the last years, the rates of multidrug-resistant (MDR) Gram-negative bacilli (GNB) are increasing worldwide (4, 5). This is particularly frequent in cancer patients due to recurrent hospital admissions and common antibiotic use driving to selective pressure (6 – 8). In this scenario, antibiotic coverage is challenging and inappropriate empirical antibiotic treatment (IEAT) has been associated with increased mortality (9, 10).

Malignant biliary obstruction commonly requires biliary drainage and/or biliary stent placement. Chronic anatomical disruption of the biliary tract, biliary stents, and frequent biliary tract manipulations render these patients highly susceptible to recurrent episodes of biliary source infections (11). Secondary complications, such as hepatic abscesses, are also frequent. These frequent and recurrent infections are associated with delayed or even discontinued chemotherapy treatments further contributing to the poor prognosis of these patients (12).

Currently, there is scarce information dealing with the characteristics and outcomes of biliary source infections in oncological patients. We aimed to describe the characteristics and outcomes of biliary source BSIs in oncological patients. Secondarily, we analyzed risk factors for MDR isolates, recurrent episodes, and mortality.

## MATERIALS AND METHODS

### Setting, study population, and design

This study was performed at Hospital Clinic in Barcelona (Spain), an 800-bed university tertiary center that provides broad and specialized medical, surgical, and intensive care attention for an urban adult population of 500,000 people. The oncology service at the Hospital Clinic provides care for a wide range of tumors, with the exception of certain rare sarcomas, and is the tertiary reference center for five regional hospitals. Additionally, its clinical trial unit also receives patients from different parts of the country and occasionally from overseas. A specific database prospectively collected all episodes of bacteremia occurring in oncological patients. For this study, we retrospectively analyzed all episodes of biliary source BSI in adults (>18 yr), from 2008 to 2019. The following data were recorded: age and gender, comorbidities, solid neoplasm and its characteristics, site of acquisition, predisposing factors and clinical conditions, causative agents and their susceptibility profile, empirical and definitive antibiotic treatment, shock at onset, ICU requirement, biliary drainage, oncological treatment delay, and mortality.

### Definitions

Definitions of comorbidities and sites of infection have been previously provided (5). Neutropenia was defined as an absolute neutrophil count of <500 cells/mm^3^. Antibiotic therapy at BSI onset was defined as the use of any antibiotic at the time of BSI. Biliary tract manipulation was considered as any surgery involving the biliary tract or the performance of an endoscopic retrograde cholangiopancreatography (ERCP) or a percutaneous transhepatic biliary cholangiography (PTC). Prior biliary source BSI was considered when it occurred during the year before the current episode. Prior admission due to suspected biliary source infection was also considered when occurring during the last year, was based on clinical judgment, and implied the absence of BSI. This was retrospectively assessed by investigators (I.G., M.C., and P.P.-A.) based on the discharge report and the available complementary tests. Septic shock was defined as those episodes of sepsis requiring the use of vasopressors due to persistent hypotension despite fluid therapy (13). IEAT was reported when the empirical therapy did not include at least one *in vitro* active antibiotic against the isolated microorganism. Hepatic abscesses were considered to be secondary to the BSI episode when they occurred within the following few weeks. Oncological treatment delay was retrospectively assessed with the treating oncologists. The following GNB were classified as MDR: (i) third-generation cephalosporin resistant Enterobacterales; (ii) carbapenem resistant Enterobacterales; and (iii) non-fermenting GNB resistant to at least three classes of antibiotics: carbapenems, ureidopenicillins, cephalosporins (ceftazidime and cefepime), monobactams, aminoglycosides, and fluoroquinolones (14). Mortality was defined as death by any cause within the first 30 d of BSI onset.

### Microbiological methods

Blood samples were processed using the BACTEC 9240 system or Bactec FX system (Becton-Dickinson Microbiology Systems), with an incubation period of 5 d. Isolates were identified using MALDI-TOF mass spectrometry, which was supplemented with biochemical reactions during the first 2 yr for additional confirmation. Antimicrobial susceptibility testing was performed using a microdilution system (Phoenix system, Becton Dickinson, Franklin Lakes, NJ) or the Etest (AB Biodisk, Solna, Sweden/bioMérieux, Mercy l’Etoile, France). ESBL-producing bacteria were suspected by MIC results and confirmed by a double-disc synergy test (15). Carbapenemase-producing Enterobacterales were phenotypically detected by the modified carbapenem inactivation method (mCIM) (16), in combination with the NG-Test CARBA 5 lateral flow immunoassay (NG Biotech, France) to detect the five most prevalent carbapenemases (KPC, OXA-48-like, VIM, IMP, and NDM) (17). Current EUCAST breakpoints for each year were used to define susceptibility or resistance to these antimicrobial agents, and intermediate susceptibility was considered as resistance.

### Statistical analysis

Categorical variables were described by counts and percentages, whereas continuous variables were expressed as means and standard deviations (SDs) or medians and interquartile ranges (IQRs). The Chi-square Pearson test and the Mann-Whitney U test or the *t*-student test were used to compare the distribution of categorical and continuous variables, respectively. Three different multivariable regression models (step-forward procedure) were used to identify the independent risk factors for (i) BSI caused by MDR pathogens, (ii) recurrent biliary source BSI, and (iii) mortality. The goodness of fit of the multivariate model was assessed by the Hosmer-Lemeshow test and the area under the receiver operating characteristic (ROC) curve. The threshold for statistical significance was defined as a two-tailed *P* < 0.05. A rule was developed to classify patients into different risk groups for recurrent BSIs based on the comparison between patients with recurrent episodes of biliary source BSIs and those who had only one episode. Resulting beta-coefficients of significant predictors of recurrent biliary source BSIs were used to assign a value (risk score) to each variable. This was achieved using a score chart approach. We then divided by the smallest coefficient, which by definition had a score of 1. The other predictors got rounded scores (18). The scores’ accuracy was assessed by the area under the ROC curve. All analyses were performed using SPSS software (version 25.0; SPSS, Inc., Chicago, IL).

## RESULTS

### Cohort and clinical characteristics of BSI episodes

Over the study period, 400 biliary source BSIs in 291 oncological patients were documented. Table 1 describes demographic features, comorbidities, and neoplasia characteristics of the cohort. The most frequent neoplasms were pancreas (35.8%), biliary (30%), and colorectal (15.8%) cancers. Biliary tract compromise was secondary to primary neoplasia in 62.7% while metastatic in the remainder.

**TABLE 1:** Clinical and demographic characteristics of patients with BSI episodes presenting with and without septic shock

	Episodes, *N* = 400 (%)
Demographics	
Male sex	265 (66.3)
Age, median (IQR)^*a*^ years	68 (60–75)
Comorbid conditions	
Diabetes mellitus	80 (20)
Chronic heart disease	23 (5.8)
Chronic liver disease	19 (4.8)
Chronic obstructive pulmonary disease	13 (3.3)
Any comorbidity	153 (38.3)
Solid neoplasm^*b*^	
Pancreas	143 (35.8)
Biliary	120 (30)
Colorectal	63 (15.8)
Hepatocellular carcinoma	18 (4.5)
Gastric	15 (3.8)
Breast cancer	12 (3)
Other	45 (11.3)
Specific oncological treatment	133 (33.3)
Disease status evaluation	
Not evaluable	241 (60.3)
Progression	105 (26.3)
Stable disease	43 (10.8)
Response	11 (2.8)
Biliary tract compromise	
Primary	251 (62.7)
Metastatic	149 (37.3)

^*a*^ IQR, interquartile range.

^*b*^ There were 16 patients who had two different solid neoplasms.

Table 2 shows the clinical characteristics of the BSI episodes. Half of the patients had biliary manipulation in the last month, 60.5% had a biliary prosthesis, and 37.3% had prior biliary source BSI. Biliary drainage was required in 49.5% episodes, and 12% developed secondary hepatic abscesses. Oncological treatment was delayed in 110 (27.5%) patients and stopped in 42 (10.5%) additional cases after BSI episodes.

**TABLE 2:** Biliary tract manipulation and clinical characteristics at BSI onset

	Episodes, *N* = 400 (%)
Predisposing factors and clinical conditions	
Previous admission (last month)	204 (51)
Previous surgery (last month)	48 (12)
Central venous catheter	124 (31)
Fever	371 (92.8)
Corticosteroid therapy	57 (14.2)
Neutropenia	4 (1)
Previous antibiotic therapy (last month)	223 (55.8)
Biliary stent	242 (60.5)
Previous biliary manipulation (last month)	200 (50)
Antibiotic prophylaxis prior to manipulation	136 (68)^*c*^
Prior biliary source BSI^*a*^	118 (29.5)
Number of previous biliary BSI, mean (SD)^*b*^	1.63 (0.99)^*d*^
Same bacteria previously producing BSI^*a*^	55 (46.6)^*d*^
Prior admission due to suspected biliary source infection	128 (32)
Number of previous admissions due to suspected biliary source infection, mean (SD)^*b*^	1.92 (1.19)
BSI manifestation and outcomes	
Septic shock	60 (15)
Inappropriate empirical antibiotic therapy	95 (23.8)
Biliary drainage requirement	198 (49.5)
Secondary hepatic abscess	48 (12)
Secondary oncological treatment delay	110 (27.5)
Days of oncological treatment delay, mean (SD)^*b*^	31.6 (28.8)^*e*^
Oncological treatment stopped after BSI^*a*^ episode	42 (10.5)
30-d mortality	78 (19.5)
Related mortality	50 (12.5)

^*a*^ BSI, bloodstream infection.

^*b*^ SD, standard deviation.

^*c*^ Percentage among those episodes with previous biliary manipulation.

^*d*^ Percentage among those episodes with prior biliary source BSI.

^*e*^ Among episodes with secondary oncological treatment delay.

### BSI epidemiology, MDR-GNB, and risk factors for MDR-GNB

Table 3 details organisms responsible for all episodes of biliary source BSIs. Overall, 81.5% of the episodes were caused by GNB, 19.3% by Gram-positive cocci (GPC), and 1.8% by *Candida* spp. Sixty-two BSI episodes were polymicrobial (15.5%). The most frequent causative agents were *E. coli* (41.5%), followed by *Klebsiella* spp. (27%), *Enterococcus* spp. (15%), and *Pseudomonas aeruginosa* (7.8%). Overall, 86 (21.5%) episodes were caused by MDR-GNB: 28 ESBL-producer *E. coli*, 25 ESBL-producer *Klebsiella* spp., 13 ESBL- and carbapenemase-producer *Klebsiella* spp., 12 Amp-C hyperproducer *Enterobacter* spp., nine MDR-*P. aeruginosa*, three Amp-C hyperproducer *Citrobacter* spp., and two ESBL- and carbapenemase-producer *E. coli*. Six of such episodes were polymicrobial. Table S1 displays the risk factors for MDR-GNB BSI. In multivariate analysis, prior antibiotic therapy (OR 1.924, 95% CI 1.095–3.379), previous biliary manipulation (OR 1.777, 95% CI 1.071–2.949), and prior biliary source BSI (OR 2.122, 95% CI 1.251–3.600) were independently associated with increased risk of MDR-GNB.

**TABLE 3:** Etiological microorganisms causing biliary source bloodstream infection

Microorganism	Episodes, *N* = 400 (%)
Gram-negative bacteria^*d*^	326 (81.5)
*E. coli*	166 (41.5)
ESBL^*a*^ ^,*e*^	30 (18.1)
Carbapenem resistant^*e*,^ *^f^*	2 (1.2)
*Klebsiella* spp.	108 (27)
ESBL^*a*^ ^,*e*^	38 (35.2)
Carbapenem resistant^*e*^ ^,*f*^	13 (12)
*P. aeruginosa*	31 (7.8)
MDR^*b*^ ^,*e*^	9 (29)
Carbapenem resistant^*e*^	11 (35.5)
*Enterobacter* spp.	24 (6)
Amp-C hyperproducer^*e*^	12 (50)
*Citrobacter* spp.	9 (2)
Amp-C hyperproducer^*e*^	3 (33.3)
Gram-positive bacteria	77 (19.3)
*Enterococcus* spp.	60 (15)
*E. faecalis* ^*e*^	14 (23.7)
*E. faecium* ^*e*^	42 (71.2)
*E. faecium* vancomycin-resistant^*h*^	3 (7.3)
*Streptococcus* spp.	14 (3.5)
*S. aureus*	1 (0.3)
MRSA^*c,e*^	1 (100)
Candidemia	7 (1.8)
Polymicrobial	62 (15.5)
MDR-GNB^*c*^ *^,^* ^*g*^	86 (21.5)
Any MDR^*b*^ *^,^* ^*g*^	89 (22.3)

^*a*^ ESBL, extended-spectrum beta-lactamase.

^*b*^ MDR, multidrug resistant.

^*c*^ MRSA, methicillin-resistant *S. aureus.*

^*d*^ GNB, gram-negative bacilli.

^*e*^ Percentage among their species.

^*f*^ All isolates were also ESBL-producers.

^*g*^ Including six polymicrobial episodes caused by two different MDR isolates.

^*h*^ Percentage among *E. faecium* isolates.

**Fig 1:**
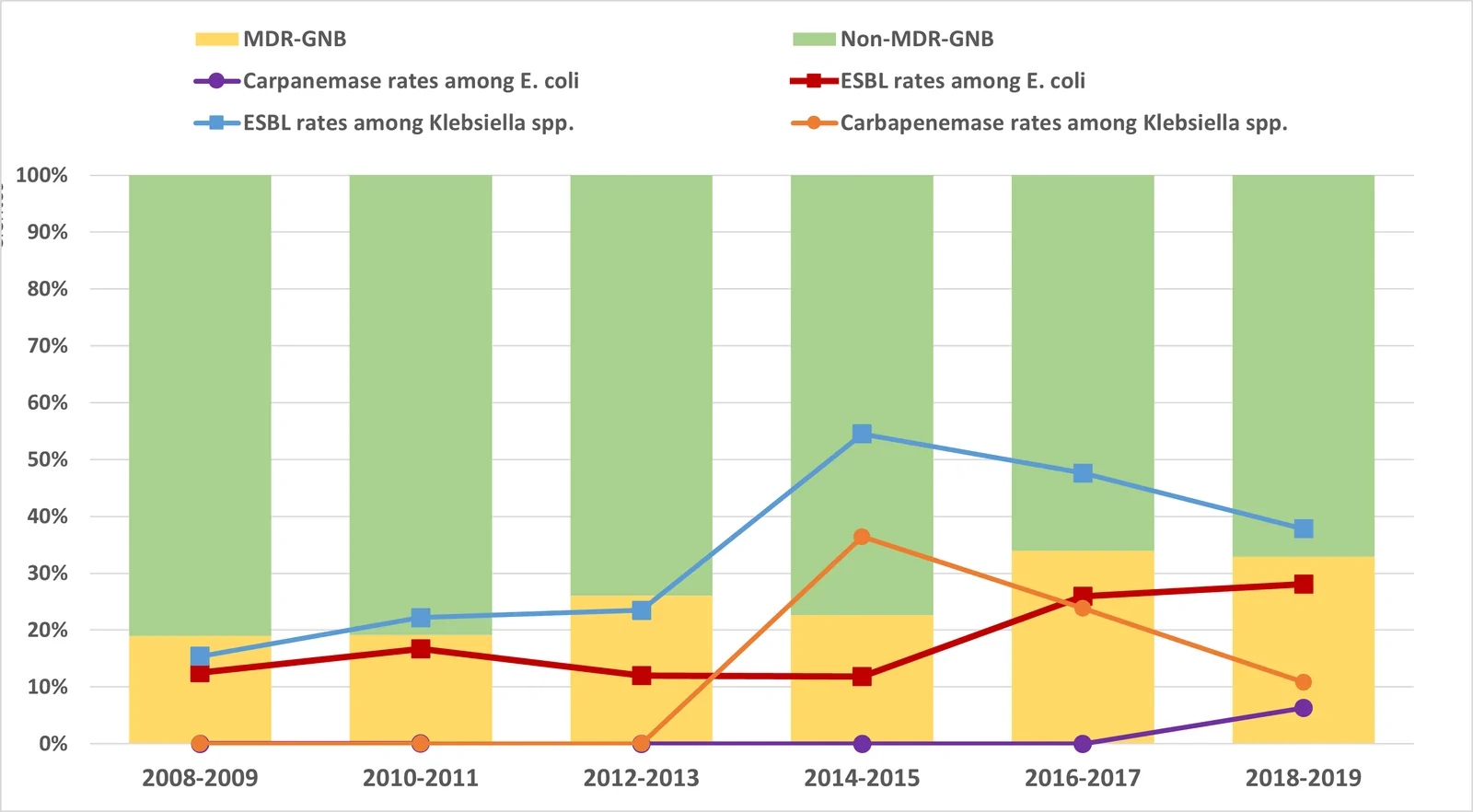
Evolution of multidrug resistance among Gram-negative bacilli and ESBL and carbapenemase producers among *E. coli* and *Klebsiella* spp. MDR, multidrug resistant; GNB, Gram-negative bacilli; ESBL, extended-spectrum beta-lactamase.

Figure 1 shows the rates over time of MDR isolates among BSI episodes caused by GNB and the rates of ESBL and carbapenemase producers among episodes caused by *E. coli* and *Klebsiella* spp. The rates of MDR-GNB increased over time (*P* value for trends <0.001).

### Recurrent episodes

There were 73 patients who later developed a new episode of BSI, for a total of 118 recurrent BSI episodes (Table 2). These 73 patients with recurrent episodes were compared with the 209 patients who presented just one episode of BSI to identify risk factors for recurrence. Table S2 shows the univariate and multivariate analysis of risk factors for recurrent biliary source BSIs. In multivariate analysis, prior antibiotic therapy (OR 3.781, 95% CI 1.906–7.503), biliary prosthesis (OR 2.232, 95% CI 1.157–4.305), prior admission due to suspected biliary source infection (OR 4.409, 95% CI 2.338–8.311), and BSI episode caused by an MDR-GNB (OR 2.857, 95% CI 1.389–5.874) were associated with increased risk of recurrent biliary source BSIs.

Results from the multivariate analysis were used to establish the clinical prediction rule. Per regression coefficients, 1 point was assigned to each parameter, except for those of prior antibiotic therapy, and prior admission due to suspected biliary source infection (2 points each). The discriminatory power of the clinical prediction rule—as evaluated by the area under the ROC curve—was 0.819 (95% CI 0.766–0.874), demonstrating a strong ability to predict the risk of recurrent biliary source BSIs. Table 4 details various cutoff points with different degrees of sensitivity, specificity, and predictive values for prediction optimization per clinical setting.

**TABLE 4:** Sensitivity, specificity, and predictive values of different operating cutoff points for predicting relapsing biliary source bloodstream infections

Score	*N*	Sensitivity	Specificity	PPV^*a*^	NPV^*b*^
>0	247	97.1%	19.2%	26.7%	95.6%
>1	201	97.1%	39.7%	32.8%	97.8%
>2	161	91.2%	55.8%	38.5%	95.4%
>3	84	70.6%	79.5%	51.1%	89.9%
>4	70	57.4%	86.2%	55.8%	86.9%
>5	22	22.1%	96.9%	68.4%	80.4%
>6	8	8.8%	99.1%	74.8%	78.2%
>7	1	1.5%	100%	100%	77%

^*a*^ PPV, positive predictive value.

^*b*^ NPV, negative predictive value.

### IEAT and mortality

IEAT was administered in 95 (23.8%) episodes and was more frequent among MDR-GNB (47.7% vs 17.2%, *P* < 0.001). Overall, 30-d mortality was 19.5%, with 12.5% of related mortality. Mortality was higher in episodes receiving IEAT (27.4% vs 17%, *P* = 0.027). Table S3 shows the univariate and multivariate analysis of risk factors for mortality. In multivariate analysis, female sex (OR 2.503, 95% CI 1.474–4.249), septic shock (OR 4.355, 95% CI 2.319–8.182), IEAT (OR 2.255, 95% CI 1.262–4.030), and BSI caused by *Klebsiella* spp. (OR 1.807, 95% CI 1.035–3.157) were independently associated with increased mortality.

## DISCUSSION

The current study describes a large cohort of oncological patients with biliary source BSIs. The most important findings were (i) oncological treatment was commonly delayed and even stopped after biliary source BSI episodes, (ii) most BSI episodes were caused by GNB, especially *E. coli* and *Klebsiella* spp., and MDR-GNB were frequent (>20%) and significantly increased over time; (iii) prior antibiotic therapy, prior biliary manipulation, and prior biliary source BSI were associated with increased risk of MDR-GNB; (iv) over a quarter of patients presented recurrent BSI episodes, and this was particularly frequent in patients receiving prior antibiotic therapy, with biliary prosthesis, prior admissions due to suspected biliary source infections, and BSI episodes caused by MDR-GNB; (v) a simple prediction score assigning 1 point to biliary prosthesis and MDR-GNB episodes and 2 points to prior antibiotic therapy and prior admission due to suspected biliary source infection identified those patients at risk of relapsing episodes with a discriminatory power of 0.819; (vi) a large proportion (23.8%) of patients received IEAT, especially in those episodes caused by MDR-GNB; and (vii) 30-d mortality was high (19.5%) and was independently associated with female sex, septic shock, IEAT, and BSI caused by *Klebsiella* spp.

Biliary source BSI is a frequent complication in patients with malignant biliary obstruction (1, 9). Additionally, in our study, oncological treatment was commonly delayed and even stopped following the BSI episode. Apart from directly related mortality, which was already important (12.5%), delays in specific treatment may further impact the survival of these patients, which is already poor and highly dependent on the chemotherapeutic (19, 20).

In our cohort, biliary source BSI were mostly caused by GNB (>80%), in particular by Enterobacterales. Additionally, over 20% of episodes were caused by MDR-GNB, and these episodes significantly increased over time, especially due to the increase in ESBL isolates, but also due to the appearance of carbapenem resistant *Klebsiella* spp. Prior series of biliary source BSIs in oncological patients reported similar causative agents but with significantly lower rates of MDR-GNB of around 10% and no reporting of carbapenem resistant Enterobacterales (21, 22). We found that prior antibiotic therapy, previous biliary manipulation, and prior biliary source BSI were independent risk factors for MDR-GNB. These findings have been previously described (22) and are not unexpected since they drive healthcare-related transmission (manipulation) and selection (prior antibiotic treatment) of MDR isolates.

Recurrent biliary source BSI is a major problem in oncological patients with biliary tract compromise. In our cohort, over a quarter of patients developed a recurrent BSI episode, with many patients presenting over three recurrent BSIs. Some previous studies reported that the main risk factors for recurrent acute cholangitis were residual stones, bile duct stricture, percutaneous biliary procedures, stent placement, and tumoral obstruction (23, 24). However, specific information on oncological patients is missing. In our study, prior antibiotic therapy and BSI caused by MDR-GNB were associated with recurrence. These factors are probably the cause (antibiotic consumption) and consequence (MDR-GNB causing the infection) of a profoundly disrupted gut microbiota composition, leading to the predominance of pathogenic species and increasing the risk of a new BSI episode (25, 26). A biliary prosthesis was also found to be predictive of recurrence. Foreign materials are commonly associated with biofilm formation leading to persistent and recurrent infections by bacteria that cannot be eradicated without prosthesis removal (11, 27). The fact that almost 50% of the recurrent BSI episodes were caused by the same bacteria would support the presence of bacterial biofilm on the prosthesis surface not eradicated with the standard antibiotic treatment. Due to the high morbidity and mortality associated with these recurrent infections, we developed a score to be able to predict which patients are more likely to recur after a biliary source BSI episode. This score needs to be prospectively validated but could potentially be used to establish prophylactic antibiotic regimens to reduce recurrences. However, this is challenging due to the high rates of MDR infections.

In our scenario of high rates of MDR-GNB, almost a quarter of patients received IEAT. Additionally, mortality was high (≈20%), particularly in episodes receiving IEAT and episodes caused by *Klebsiella* spp. These rates of IEAT and mortality are very similar to those previously described in cancer patients with biliary source BSI (21, 22). A thorough knowledge of BSI epidemiology is essential to establish effective empirical treatments and decrease mortality (10, 28). Since the risk factors for MDR-GNB were somewhat unspecific, we believe a wise approach would be to decide the broadness of empirical treatment based on patients’ severity. In our setting, with such high rates of ESBL producing Enterobacterales, empirical treatment with carbapenems seems logical. However, due to the increasing rates of carbapenem resistance, ceftazidime-avibactam should be considered in severely sick or septic shock patients.

The strengths of this study are the prospective and thorough collection of most data by a senior specialist evaluating every clinical and microbiological data and the large number of biliary source BSIs identified in oncological patients. However, some limitations should be acknowledged. First, our study was conducted at a single center, in a particular geographical area. Different microbiology and drug resistance patterns are expected in other areas. Second, the denominator of patients at risk of biliary source BSI related to a baseline malignancy was not available. Therefore, the real incidence of these infections could not be estimated. Third, for those recurrent episodes caused by the same pathogen, no genomic analysis was available to evaluate whether the new infection was caused or not by the same strain. Fourth, only those episodes with BSI were included in the analysis. However, many of these patients have recurrent fever episodes with no positive microbiological result. Although a high proportion of such cases are likely to be infectious, there exist many other factors potentially causing fever such as transfusions, drug-related reactions, catheter-related infections, etc. Additionally, some pathogens are difficult to culture, leading to their potential underrepresentation. Lastly, the score has not been validated yet, and there is a potential risk of overfitting.

In conclusion, biliary source BSI causes important morbidity in oncological patients. Most episodes are caused by GNB, with high rates of MDR isolates, which are indeed increasing. In this context, IEAT is frequently administered and mortality is high. Recurrent biliary source BSIs are very common. A simple score to identify recurrent episodes was developed to potentially establish prophylactic strategies in high-risk patients.

## Data Availability

The utilized database can be accessed in Zenodo.

## SUPPLEMENTAL MATERIAL

Supplemental Tables spectrum.02142-23-s0001.docx Tables showing univariate and multivariate analyses.

ASM does not own the copyrights to Supplemental Material that may be linked to, or accessed through, an article. The authors have granted ASM a non-exclusive, world-wide license to publish the Supplemental Material files. Please contact the corresponding author directly for reuse.
