# Supplementary material for: Epidemiology and risk factors for recurrence in biliary source bloodstream infection episodes in oncological patients
Source: Microbiol Spectr. 2023 Aug 23;11(5):e02142-23. doi: 10.1128/spectrum.02142-23 (PMC10580831; doi:10.1128/spectrum.02142-23)
Supplement: Supplemental Tables — Tables showing univariate and multivariate analyses. [file spectrum.02142-23-s0001.docx]

**Supplementary material. Epidemiology and risk factors for recurrence in biliary source bloodstream infection episodes in oncological patients.**

**Supplementary Table S1. Univariate and multivariate risk factor for bloodstream infection caused by MDR-GNB.**

|  | Non-MDR-GNB  N=314 (%) | MDR-GNB  N=86 (%) | Univariate OR  (95% CI) | P value | Multivariate OR (95% CI) | P value |
| --- | --- | --- | --- | --- | --- | --- |
| Male sex | 206 (65.6) | 59 (68.6) | 1.15 (0.69-1.91) | 0.602 | - | - |
| Any comorbidity | 125 (39.8) | 28 (32.6) | 0.73 (0.44-1.21) | 0.220 | - | - |
| Pancreas cancer | 115 (36.6) | 28 (32.6) | 0.84 (0.50-1.39) | 0.486 | - | - |
| Biliary tract cancer | 94 (29.9) | 26 (30.2) | 1.01 (0.60-1.71) | 0.958 | - | - |
| Colorectal cancer | 44 (14.0) | 19 (22.1) | 1.74 (0.95-3.17) | 0.068 | - | - |
| Hepatocellular carcinoma | 14 (4.5) | 4 (4.7) | 1.05 (0.34-3.27) | 1.000 | - | - |
| Gastric cancer | 13 (4.1) | 2 (2.3) | 0.55 (0.12-2.49) | 0.748 | - | - |
| Specific oncological treatment | 105 (33.5) | 28 (32.6) | 0.96 (0.58-1.59) | 0.863 | - | - |
| Metastatic biliary tract compromise | 114 (36.3) | 35 (40.7) | 1.20 (0.74-1.96) | 0.455 | - | - |
| Previous admission | 150 (47.8) | 54 (62.8) | 1.85 (1.13-3.01) | **0.014** | 1.28 (0.75-2.20) | 0.368 |
| Previous surgery | 38 (12.1) | 10 (11.6) | 0.96 (0.46-2.01) | 0.905 | - | - |
| Corticosteroid therapy | 48 (15.3) | 9 (10.5) | 0.65 (0.30-1.38) | 0.257 | - | - |
| Neutropenia | 4 (1.3) | 0 (0.0) | - | 0.582 | - | - |
| Prior antibiotic therapy | 160 (51.0) | 63 (73.3) | 2.64 (1.56-4.46) | **<0.001** | 1.92 (1.10-3.38) | **0.023** |
| Biliary stent | 187 (59.6) | 55 (64) | 1.21 (0.74-1.98) | 0.460 | - | - |
| Previous biliary tract manipulation | 146 (46.5) | 54 (62.8) | 1.94 (1.19-3.17) | **0.007** | 1.78 (1.07-2.95) | **0.026** |
| Prior biliary source bloodstream infection | 78 (24.8) | 40 (46.5) | 2.63 (1.60-4.32) | **<0.001** | 2.12 (1.25-3.60) | **0.005** |
| Prior admission due to suspected biliary source infection | 102 (32.5) | 26 (30.2) | 0.90 (0.54-1.51) | 0.692 | - | - |
| Septic shock | 50 (15.9) | 10 (11.6) | 0.70 (0.34-1.44) | 0.323 | - | - |
| Polymicrobial bloodstream infection | 46 (14.6) | 16 (18.6) | 1.33 (0.71-2.49) | 0.369 | - | - |

*Abbreviations. MDR: multidrug resistant; GNB: Gram-negative bacilli; OR: odds ratio; CI: confidence interval.

**Supplementary Table S2. Univariate and multivariate analysis of risk factors for recurrent biliary source bloodstream infection.**

|  | Non-recurrent N=209 (%) | Recurrent N=73 (%) | Univariate OR  (95% CI) | P value | Multivariate OR (95% CI) | P value |
| --- | --- | --- | --- | --- | --- | --- |
| Male sex | 133 (63.6) | 48 (65.8) | 1.10 (0.63-1.92) | 0.745 | - | - |
| Age >65 years | 123 (58.9) | 45 (61.6) | 1.12 (0.65-1.94) | 0.676 | - | - |
| Any comorbidity | 98 (46.9) | 25 (34.2) | 0.59 (0.34-1.03) | 0.061 | - | - |
| Pancreas cancer | 72 (34.4) | 28 (38.4) | 1.18 (0.68-2.06) | 0.548 | - | - |
| Biliary tract cancer | 67 (32.1) | 22 (30.1) | 0.91 (0.51-1.63) | 0.761 | - | - |
| Colorectal cancer | 21 (10.0) | 13 (17.8) | 1.94 (0.92-4.11) | 0.080 | - | - |
| Hepatocellular carcinoma | 11 (5.3) | 2 (2.7) | 0.51 (0.11-2.34) | 0.525 | - | - |
| Gastric cancer | 7 (3.3) | 3 (4.1) | 1.24 (0.31-4.91) | 0.722 | - | - |
| Breast cancer | 9 (4.3) | 1 (1.4) | 0.31 (0.04-2.45) | 0.462 | - | - |
| Specific oncological treatment | 58 (27.9) | 26 (35.6) | 1.43 (0.81-2.52) | 0.214 | - | - |
| Metastatic biliary tract compromise | 67 (32.1) | 29 (39.7) | 1.40 (0.81-2.43) | 0.234 | - | - |
| Previous admission | 86 (41.1) | 47 (64.4) | 2.59 (1.49-4.49) | **0.001** | 1.32 (0.67-2.61) | 0.428 |
| Corticosteroid therapy | 33 (15.8) | 13 (17.8) | 1.16 (0.57-2.34) | 0.688 | - | - |
| Prior antibiotic therapy | 91 (43.5) | 58 (79.5) | 5.01 (2.67-9.42) | **<0.001** | 3.78 (1.91-7.50) | **<0.001** |
| Biliary stent | 106 (50.7) | 53 (72.6) | 2.58 (1.44-4.61) | **0.001** | 2.23 (1.16-4.31) | **0.017** |
| Previous biliary tract manipulation | 99 (47.4) | 35 (47.9) | 1.02 (0.60-1.75) | 0.932 | - | - |
| Prior admission due to suspected biliary source infection | 46 (22.0) | 43 (58.9) | 5.08 (2.87-8.98) | **<0.001** | 4.41 (2.34-8.31) | **<0.001** |
| Septic shock | 38 (18.2) | 7 (9.6) | 0.48 (0.20-1.12) | 0.084 | - | - |
| Secondary hepatic abscess | 19 (9.1) | 14 (19.2) | 2.37 (1.12-5.02) | **0.021** | 1.99 (0.82-4.87) | 0.130 |
| Secondary oncological treatment delay | 38 (18.2) | 26 (35.6) | 2.49 (1.37-4.51) | **0.002** | 1.87 (0.94-3.72) | 0.074 |
| Gram-negative bacili | 173 (82.8) | 62 (84.9) | 1.17 (0.56-2.45) | 0.670 | - | - |
| *E. coli* | 80 (38.3) | 30 (41.1) | 1.13 (0.65-1.94) | 0.671 | - | - |
| *Klebsiella* spp. | 61 (29.2) | 17 (23.3) | 0.74 (0.40-1.37) | 0.332 | - | - |
| *P. aeruginosa* | 17 (8.1) | 8 (11.0) | 1.39 (0.57-3.37) | 0.465 | - | - |
| *Enterobacter* spp. | 12 (5.7) | 6 (8.2) | 1.47 (0.53-4.07) | 0.456 | - | - |
| *Enterococcus* spp. | 25 (12.0) | 11 (15.1) | 1.31 (0.61-2.81) | 0.493 | - | - |
| *Streptococcus* spp. | 10 (4.8) | 1 (1.4) | 0.28 (0.04-2.20) | 0.299 | - | - |
| Candidemia | 4 (1.9) | 2 (2.7) | 1.44 (0.26-8.05) | 0.651 | - | - |
| Polymicrobial | 35 (16.7) | 6 (8.2) | 0.45 (0.18-1.11) | 0.075 | - | - |
| MDR-GNB | 31 (14.8) | 24 (32.9) | 2.81 (1.51-5.23) | **0.001** | 2.86 (1.39-5.87) | **0.004** |

*Abbreviations. OR: odds ratio; CI: confidence interval; MDR: multidrug resistant; GNB: Gram-negative bacilli.

**Supplementary Table S3. Univariate and multivariate analysis of risk factors for mortality.**

|  | Alive  N=322 (%) | Dead  N=78 (%) | Univariate OR  (95% CI) | P value | Multivariate OR  (95% CI) | P value |
| --- | --- | --- | --- | --- | --- | --- |
| Female sex | 96 (29.8) | 39 (50.0) | 2.35 (1.42-3.90) | **0.001** | 2.50 (1.47-4.25) | **0.001** |
| Age >65 years | 193 (59.9) | 44 (56.4) | 0.87 (0.53-1.43) | 0.569 | - | - |
| Diabetes mellitus | 65 (20.2) | 15 (19.2) | 0.94 (0.50-1.76) | 0.850 | - | - |
| Chronic heart disease | 17 (5.3) | 6 (7.7) | 1.50 (0.57-3.93) | 0.411 | - | - |
| Chronic liver disease | 15 (4.7) | 4 (5.1) | 1.11 (0.36-3.43) | 0.773 | - | - |
| Chronic obstructive pulmonary disease | 12 (3.7) | 2 (2.6) | 0.68 (0.15-3.10) | 1.000 | - | - |
| Any comorbidity | 127 (39.4) | 26 (33.3) | 0.77 (0.46-1.29) | 0.319 | - | - |
| Pancreas cancer | 118 (36.6) | 25 (32.1) | 0.82 (0.48-1.38) | 0.447 | - | - |
| Biliary tract cancer | 93 (28.9) | 27 (34.6) | 1.30 (0.77-2.20) | 0.321 | - | - |
| Colorectal cancer | 54 (16.8) | 9 (11.5) | 0.647 (0.31-1.38) | 0.255 | - | - |
| Hepatocellular carcinoma | 16 (5.0) | 2 (2.6) | 0.50 (0.11-2.24) | 0.545 | - | - |
| Gastric cancer | 13 (4.0) | 2 (2.6) | 0.63 (0.14-2.83) | 0.745 | - | - |
| Breast cancer | 11 (3.4) | 1 (1.3) | 0.367 (0.05-2.89) | 0.475 | - | - |
| Specific oncological treatment | 107 (33.3) | 26 (33.3) | 1.00 (0.59-1.69) | 1.000 | - | - |
| Metastatic biliary tract compromise | 121 (37.6) | 28 (35.9) | 0.93 (0.56-1.56) | 0.783 | - | - |
| Previous admission | 166 (51.6) | 38 (48.7) | 0.89 (0.54-1. 46) | 0.653 | - | - |
| Corticosteroid therapy | 42 (13.0) | 15 (19.2) | 1.59 (0.83-3.04) | 0.161 | - | **-** |
| Neutropenia | 4 (1.2) | 0 (0.0) | 0.80 (0.77-0.84) | 1.000 | - | **-** |
| Prior antibiotic therapy | 180 (55.9) | 43 (55.1) | 0.97 (0.59-1.59) | 0.902 | - | - |
| Biliary stent | 201 (62.4) | 41 (52.6) | 0.67 (0.41-1.10) | 0.110 | - | - |
| Previous biliary tract manipulation | 164 (50.9) | 36 (46.2) | 0.83 (0.50-1.36) | 0.449 | - | - |
| Prior biliary source BSI | 93 (28.9) | 25 (32.1) | 1.16 (0.68-1.98) | 0.582 | - | - |
| Prior admission due to suspected biliary source infection | 99 (30.7) | 29 (37.2) | 1.33 (0.80-2.24) | 0.274 | - | **-** |
| Septic shock | 36 (11.2) | 24 (30.8) | 3.53 (1.95-6.39) | **<0.001** | 4.36 (2.32-8.18) | **<0.001** |
| Inappropriate empirical antibiotic therapy | 69 (21.4) | 26 (33.3) | 1.83 (1.07-3.15) | **0.027** | 2.26 (1.26-4.03) | **0.006** |
| Biliary drainage requirement | 165 (51.2) | 33 (42.3) | 0.70 (0.42-1.15) | 0.157 | - | **-** |
| Secondary hepatic abscess | 36 (11.2) | 12 (15.4) | 1.44 (0.71-2.93) | 0.305 | - | - |
| Secondary oncological treatment delay | 93 (28.9) | 17 (21.8) | 0.67 (0.38-1.24) | 0.208 | - | - |
| Gram-negative bacilli | 259 (80.4) | 67 (85.9) | 1.48 (0.74-2.97) | 0.265 | - | - |
| *E. coli* | 134 (41.6) | 32 (41.0) | 0.98 (0.59-1.61) | 0.925 | - | - |
| ESBL *E. coli* | 25 (7.8) | 5 (6.4) | 0.81 (0.30-2.20) | 0.814 | - | - |
| *Klebsiella* spp. | 80 (24.8) | 28 (35.9) | 1.69 (1.00-2.87) | **0.049** | 1.81 (1.04-3.16) | **0.038** |
| ESBL *Klebsiella* spp. | 26 (8.1) | 12 (15.4) | 2.07 (0.99-4.31) | **0.048** |  |  |
| Carbapenem resistant *Klebsiella* spp. | 8 (2.5) | 5 (6.4) | 2.69 (0.86-8.46) | 0.144 | - | - |
| *P. aeruginosa* | 25 (7.8) | 6 (7.7) | 0.99 (0.39-2.50) | 0.983 | - | - |
| MDR *P. aeruginosa* | 6 (1.9) | 3 (3.8) | 2.11 (0.52-8.62) | 0.386 | - | - |
| Carbapenem resistant *P. aeruginosa* | 9 (2.8) | 2 (2.6) | 0.92 (0.19-4.32) | 1.000 | - | - |
| *Enterobacter* spp. | 20 (6.2) | 4 (5.1) | 0.82 (0.27-2.46) | 1.000 | - | - |
| ESBL *Enterobacter* spp. | 10 (3.1) | 2 (2.6) | 0.82 (0.18-3.83) | 1.000 | - | - |
| *Citrobacter* spp. | 6 (1.9) | 3 (3.8) | 2.11 (0.56-8.62) | 0.386 | - | - |
| *Enterococcus* spp. | 53 (16.5) | 7 (9.0) | 0.50 (0.22-1.15) | 0.097 | - | - |
| *E.* *faecalis* | 13 (4.0) | 1 (1.3) | 0.31 (0.04-2.40) | 0.321 | - | - |
| *E.* *faecium* | 36 (11.2) | 6 (7.7) | 0.66 (0.27-1.63) | 0.367 | - | - |
| *Streptococcus* spp. | 14 (4.3) | 0 (0.0) | 0.80 (0.76-0.84) | 0.082 | - | - |
| Candidemia | 4 (1.2) | 3 (3.8) | 3.18 (0.70-14.51) | 0.138 | - | - |
| Polymicrobial | 49 (15.2) | 13 (16.7) | 1.11 (0.57-2.18) | 0.751 | - | - |
| MDR-GNB | 65 (20.2) | 21 (26.9) | 1.46 (0.82-2.58) | 0.194 | - | - |

*Abbreviations. OR: odds ratio; CI: confidence interval; BSI: bloodstream infection; MDR: multidrug resistant; GNB: Gram-negative bacilli.
